# Predictive model of castration resistance in advanced prostate cancer by machine learning using genetic and clinical data: KYUCOG-1401-A study

**DOI:** 10.1038/s44276-024-00093-3

**Published:** 2024-09-09

**Authors:** Masaki Shiota, Shota Nemoto, Ryo Ikegami, Shuichi Tatarano, Toshiyuki Kamoto, Keita Kobayashi, Hideki Sakai, Tsukasa Igawa, Tomomi Kamba, Naohiro Fujimoto, Akira Yokomizo, Seiji Naito, Masatoshi Eto

**Affiliations:** 1 Department of Urology, Graduate School of Medical Sciences, Kyushu University, Fukuoka, Japan; 2 Industrial & Digital Business Unit, Hitachi, Ltd., Tokyo, Japan; 3 Department of Urology, Graduate School of Medical and Dental Sciences, Kagoshima University, Kagoshima, Japan; 4 Department of Urology, Faculty of Medicine, Miyazaki University, Miyazaki, Japan; 5 Department of Urology, Graduate School of Medicine, Yamaguchi University, Ube, Japan; 6 Department of Urology, Graduate School of Biomedical Sciences, Nagasaki University, Nagasaki, Japan; 7 Department of Urology, School of Medicine, Kurume University, Kurume, Japan; 8 Department of Urology, Kumamoto University, Kumamoto, Japan; 9 Department of Urology, School of Medicine, University of Occupational and Environmental Health, Kitakyushu, Japan; 10 Department of Urology, Harasanshin Hospital, Fukuoka, Japan

## Abstract

**Background:**

The predictive power of the treatment efficacy and prognosis in primary androgen deprivation therapy (ADT) for advanced prostate cancer is not satisfactory. The objective of this study was to integrate genetic and clinical data to predict castration resistance in primary ADT for advanced prostate cancer by machine learning (ML).

**Methods:**

Clinical and single nucleotide polymorphisms (SNP) data obtained in the KYUCOG-1401-A study (UMIN000022852) that enrolled Japanese patients with advanced prostate cancer were used. All patients were treated with primary ADT. A point-wise linear (PWL) algorithm, logistic regression with elastic-net regularization, and eXtreme Gradient Boosting were the ML algorithms used in this study. Area under the curve for castration resistance and C-index for prognoses were calculated to evaluate the utility of the models.

**Results:**

Among the three ML algorithms, the area under the curve values to predict castration resistance at 2 years was highest for the PWL algorithm with all the datasets. Three predictive models (clinical model, small SNPs model, and large SNPs model) were created by the PWL algorithm using the clinical data alone, and 2 and 46 SNPs in addition to clinical data. C-indices for overall survival by the clinical, small SNPs, and large SNPs models were 0.636, 0.621, and 0.703, respectively.

**Conclusion:**

The results demonstrated that the SNPs models created by ML produced excellent prediction of castration resistance and prognosis in primary ADT for advanced prostate cancer, and will be helpful in treatment choice.

## Background

Androgen deprivation therapy (ADT) is widely used as the backbone therapy for advanced prostate cancer [1]. Current intensive therapies for metastatic prostate cancer include radiation, docetaxel, and novel androgen receptor signaling inhibitors, such as abiraterone, darolutamide, enzalutamide, and apalutamide, in addition to ADT [2]. Furthermore, triplet combination therapy, which adds such novel androgen receptor signaling inhibitors to ADT plus docetaxel, has recently been shown to prolong survival in metastatic prostate cancer [3, 4]. Therefore, prognostic estimation in ADT can help in choosing the best treatment. However, prognostic estimation in ADT using clinical parameters such as prostate-specific antigen (PSA), Gleason score, and TNM category is not satisfactory for producing C-indices of >0.7 [5–7]. Therefore, novel predictive models to more precisely estimate the response to ADT for advanced prostate cancer are needed.

Genetic background has been suggested to affect the efficacy and prognosis in ADT for prostate cancer, as indicated by different outcome among different ethnicities and consistent outcome within families [8–10]. Over the past few decades, genome wide association studies (GWAS) have discovered associations between single nucleotide polymorphisms (SNPs) and various features [11]. In a previous study, we investigated the association between the SNPs and prognosis in Japanese patients undergoing primary ADT for advanced prostate cancer by GWAS [12]. In that study, two SNPs, rs76237622 in *PRR27* and rs117573572 in *MTAP*, were validated to be associated with prognosis in ADT, but their predictive ability was not satisfactory [12].

Machine learning (ML) is a statistics-free approach that uses algorithms to identify patterns in rich and unwieldy data [13]. ML can resolve complex datasets of high dimensionality such as genomic data [14, 15]. In this study, we aimed to integrate genetic and clinical data that were obtained in the previous study [12], to predict castration resistance in primary ADT for advanced prostate cancer by ML.

## Methods

### Study population

Japanese patients with *de novo* advanced prostate cancer (TanyN1M0 or TanyNanyM1) enrolled in the KYUCOG-1401-A study (UMIN000022852) that was conducted in conjunction with a prospective multi-institutional clinical trial (KYUCOG-1401; UMIN000014243, jRCTs071180035) were included in this study. Inclusion and exclusion criteria for the KYUCOG-1401-A study have been described previously [12]. Patients (*n* = 8) censored before 2 years were excluded from this study. In the KYUCOG-1401 study, patients were randomized to receive gonadotropin-releasing hormone (GnRH) antagonist (degarelix) or agonist (leuprorelin or goserelin) plus the antiandrogen bicalutamide [16]. This study was conducted in accordance with the Declaration of Helsinki and the Japanese Ethical Guidelines for Medical and Health Research Involving Human Subjects. Eligible patients provided written informed consent. This study was approved by the Kyushu University review board (23087-00).

### Clinical data

Clinicopathological information and efficacy in treatment data were collected prospectively using an electronic data capture system, as described previously [12]. Progression was defined as PSA progression (defined as PSA level of 2.0 ng/mL or higher, a rise of 50% or more from the lowest value, and three consecutive increases in PSA measured at least one week apart) or radiographic progression, as described previously [12, 17, 18]. For the analysis of progression-free survival (PFS), cancer-specific survival (CSS), and overall survival (OS), progression or death from any cause, death from prostate cancer, and death from any cause were defined as events, respectively. Patients who did not experience any of these events were censored at the last follow-up visit. For the survival analysis, the number of days from enrollment to the earliest event or censoring date was calculated. Patients who progressed to castration resistance at 2 years were defined as non-responders and patients who did not were defined as responders. Risk stratification by J-CAPRA risk score was performed as described previously [19].

### Genetic data

Genetic data were obtained as described previously [12]. Genomic DNA was genotyped using a Japonica Array v2 according to the manufacturer’s instructions (Thermo Fisher Scientific, Waltham, MA, USA) [20–22]. This Axiom Array was customized for the Japanese genome by the Tohoku Medical Megabank Organization. Genotype calling was conducted using Genotyping Console software v4.2 (Thermo Fisher Scientific). We used the PSA-PFS at 2years-associated 2 and 46 SNPs with *p* < 1.0 × 10^−5^ and *p* < 1.0 × 10^−4^ that were identified in a previous study [12].

### Construction of simple prediction scores

The variables were classified as binary (1 or −1) or quantitative. Quantitative variables were normalized by subtracting the mean value and dividing by the standard deviation. Missing values were set to 0.

The prediction models were constructed using three ML algorithms (point-wise linear algorithm, logistic regression with elastic-net regularization algorithm, and eXtreme Gradient Boosting) and three datasets (clinical, clinical and 2 SNPs, and clinical and 46 SNPs). Details of the clinical dataset are provided in Supplementary Table 1. The point-wise linear (PWL) algorithm is a deep learning-based algorithm that was implemented using PyTorch 1.5.1 and Python 3.7.4 [23]. The PWL algorithm uses a deep (multi-layered) neural network structure that generates a logistic regression model for each sample; i.e., a weight vector tailored to each sample. The importance of each feature is computed using its weight vector. Deep unified networks were used to construct the deep neural network in which the network layers and neurons are connected in a mesh-like structure that reduces the risk of overfitting [24]. The logistic regression with elastic-net regularization (LR) algorithm and eXtreme Gradient Boosting (XGBoost) were used to build the baseline models (implemented using scikit-learn v0.24.2, xgboost 1.0.2, and Python 3.7.4) [25]. The best hyper-parameter of each model was determined by 5-fold cross validation using the discovery cohort [23]. The prediction performance of each model was calculated by area under the curve (AUC) and evaluated using the validation cohort and the models fitted by the best hyper-parameter.

The important features to predict castration resistance were determined based on an importance score that was calculated using the weight vector of the PWL algorithm. The sample-wise importance score was calculated as described previously [26]. Importance score was defined by the rate at which a sample was ranked in the top 10% of features with sample-wise importance scores. Parameters with importance scores ≥0.1 were extracted as important features. An importance score of 0.1 indicated that at least 10% of the samples had parameters that were in the top 10% of important features. Simple prediction scores were constructed using important features and the sign of the median of sample-wise weights in those features. We used the original values for the variables in the simple prediction scores. The prediction performance of the simple prediction scores was evaluated by AUC using discovery and validation cohorts.

### Estimation of effect by genetic background among different ethnic populations

Allele frequency data were obtained from the 1000 Genomes Project (https://www.internationalgenome.org/home). Estimated effect was the sum of the value for each SNP calculated as: coefficient × 2 × (minor allele frequency) × (1 − minor allele frequency) + 2 × coefficient × (minor allele frequency)^2^.

### Statistical analyses

Statistical analyses were performed using JMP16 software (SAS Institute, Cary, NC, USA). Continuous and categorical data are presented as median with interquartile range and number with percentage, respectively. The association among the categorical data was analyzed by the chi-square test. Survival analysis was performed using the Kaplan–Meier method and log-rank test. Harrell’s C-index was calculated using Stata v18 (College Station, TX, USA) as described previously [7]. All P-values were two-sided, and *P*-values < 0.05 were considered significant for all the analyses.

## Results

### Patients assignment

A total of 119 patients were included in the study, and were divided randomly in a 7:3 ratio into discovery (*n* = 82) and validation (*n* = 37) cohorts. Clinical parameters of the patients in each cohort are provided in Supplementary Table 1. Several clinical parameters including Gleason score, extent of disease grade, PSA level, and hemoglobin value were different between non-responders and responders in the discovery cohort (Supplementary Table 1). In addition, history of cerebral infarction, total type I procollagen-N-propeptide (P1NP), white blood cell count, and neutrophil count were higher in non-responders compared with their levels in responders in the discovery cohort (Supplementary Table 1).

### Predictive ability of castration resistance by three ML algorithms using genetic and clinical data

The ability of three ML algorithms (PWL, LR, XGBoost) to predict castration resistance using genetic and clinical data was evaluated. Using only the clinical data (Supplementary Table 1) to predict castration resistance at 2 years, the AUC, sensitivity, and specificity in the discovery cohort were 0.710–0.785, 0.568–0.704, and 0.644–0.689, respectively (Table 1). In the validation cohort, AUC, sensitivity, and specificity were 0.720–0.786, 0.688–0.875, and 0.579–0.684, respectively (Table 1). When the two SNPs associated with PSA-PFS at 2 years with *p* < 1.0 × 10^−5^ were used together with the clinical parameters, AUC, sensitivity, and specificity in the discovery cohort slightly improved to 0.796–0.810, 0.700–0.754, and 0.689–0.778, respectively (Table 1). In the validation cohort, AUC, sensitivity, and specificity also slightly improved to 0.701–0.878, 0.625–0.750, and 0.684–0.789, respectively (Table 1). Finally, when the 46 SNPs associated with PSA-PFS at 2 years with *p* < 1.0 × 10^−4^ were used together with the clinical parameters, AUC, sensitivity, and specificity in greatly improved in the discovery cohort to 0.962–0.988, 0.600–0.864, and 0.978–0.978, respectively (Table 1). In the validation cohort, AUC, sensitivity, and specificity also greatly improved to 0.984–1.000, 0.875–0.938, and 1.000–1.000, respectively (Table 1).

**Table 1:** Predictive performance by machine learning methods using the indicated parameters.

Parameters	Mechine learning method	Discovery cohort^a^			Validation cohort		
		AUC	Sensitivity	Specificity	AUC	Sensitivity	Specificity
Clinical data	PWL	0.773	0.639	0.911	0.786	0.812	0.684
	LR	0.710	0.568	0.644	0.727	0.688	0.684
	XGBoost	0.785	0.704	0.689	0.720	0.875	0.579
Clinical data and 2 SNPs (*p* < 1.0 × 10^−5^)	PWL	0.810	0.754	0.778	0.878	0.750	0.789
	LR	0.801	0.704	0.756	0.862	0.750	0.842
	XGBoost	0.796	0.700	0.689	0.701	0.625	0.684
Clinical data and 46 SNPs (*p* < 1.0 × 10^−4^)	PWL	0.988	0.864	0.978	1.000	0.938	1.000
	LR	0.963	0.725	0.978	1.000	0.938	1.000
	XGBoost	0.962	0.600	0.978	0.984	0.875	1.000

*AUC* area under the curve, *PWL* point-wise linear, *LR* logistic regression with elastic-net regularization, *SNP* single nucleotide polymorphism, *XGBoost* eXtreme Gradient Boosting.

^a^Mean values by 5-fold cross validation.

### Model creation to predict castration resistance using genetic and clinical data by ML

The PWL algorithm produced the highest AUC values in the three ML algorithms in the discovery and validation cohorts, with the exception of the clinical model in the discovery cohort (Table 1). Therefore, we created a prediction model for castration resistance using genetic and clinical data by the PWL algorithms.

**Fig. 1:**
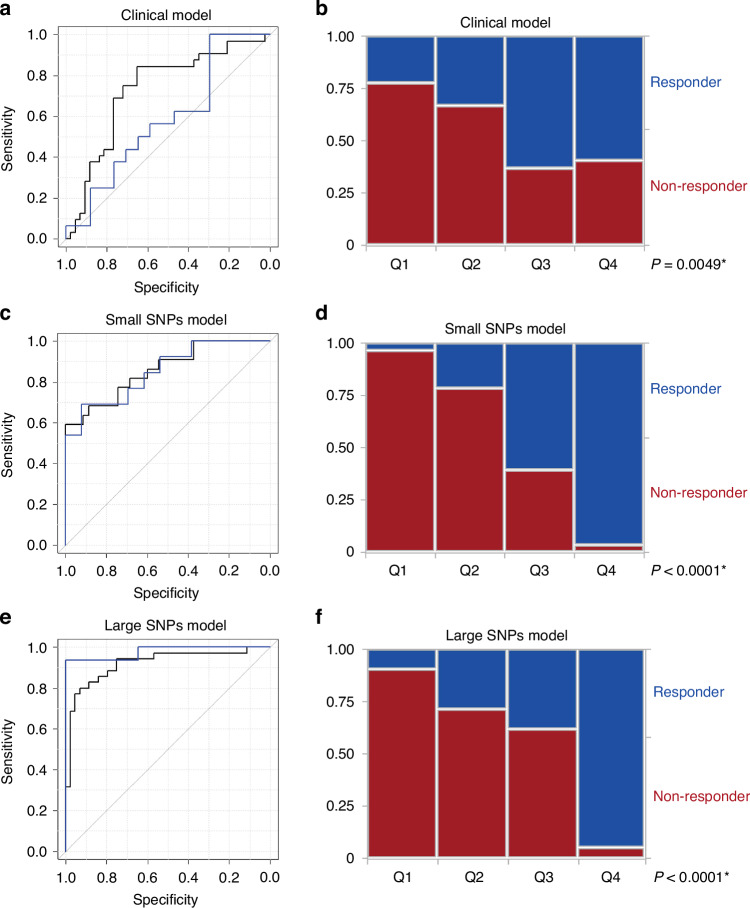
Predictive models of castration resistance at 2 years created by machine learning using clinical and genetic parameters. Receiver operating characteristic curve for castration resistance at 2 years in clinical model (**A**), small SNPs model (**C**), and large SNPs model (**E**). Distribution of predictive scores of samples among discovery and validation cohorts according to quarterly-divided groups (Q1–Q4) based on scores by clinical model (**B**), small SNPs model (**D**), and large SNPs model (**F**).

When critical parameters associated with castration resistance at 2 years were used, 12 clinical parameters were identified (Table 2). Besides the known predictive factors, Gleason score, PSA level, N-category, albumin level, and total testosterone level, other factors including comorbidity with hypertension, total cholesterol level, lymphocyte ratio, blood urea nitrogen level, comorbidity with dyslipidemia, and aspartate aminotransferase level were also identified as critical parameters (Table 2). When the predictive model (clinical model) was created using the formula in Supplementary Table 2, the AUCs in the discovery and validation cohorts were 0.730 (95% CI, 0.610–0.849) and 0.585 (95% CI, 0.383–0.787), respectively (Fig. 1A). When prediction scores calculated by the clinical model were divided quarterly (Q1–Q4), the ranges were −1.42267 to 6.86566 in Q1, 6.901361–8.656931 in Q2, 8.7444–10.01411 in Q3, and 10.06535–13.23641 in Q4. The clinical model correctly predicted 21/27 (77.8%) in Q1 and 16/27 (59.3%) in Q4 to be non-responders and responders, respectively (*p* = 0.0049, Fig. 1B).

**Table 2:** Important features in clinical model.

#	Parameter	Score	Median weight
1	Hypertension	0.776	0.012
2	Total testosterone	0.524	0.015
3	Total cholesterol	0.495	0.015
4	Lymphocyte (%)	0.356	0.012
5	Gleason score	0.337	−0.011
6	ALB	0.178	0.008
7	BUN	0.176	−0.012
8	N-category	0.171	−0.007
9	Age	0.132	0.006
10	Dyslipidemia	0.117	−0.007
11	AST	0.112	0.008
12	PSA	0.107	−0.009

*ALB* albumin, *AST* aspartate aminotransferase, *BUN* blood urea nitrogen, *PSA* prostate-specific antigen.

When the two SNPs (*p* < 1.0 × 10^−5^) were added to the clinical parameters, six clinical parameters and two SNPs were identified to be critical to predict castration resistance at 2 years (Table 3). Besides the known predictive factors, Gleason score and extent of disease grade, other factors including comorbidity with diabetes mellitus, creatine kinase level, total P1NP level, and lymphocyte ratio were also identified as critical parameters (Table 3). When the predictive model (small SNPs model) was created using the formula in Supplementary Table 2, the AUCs in the discovery and validation cohorts improved to 0.857 (95% CI, 0.756–0.959) and 0.852 (95% CI, 0.706–0.998), respectively (Fig. 1C). When scores calculated by small SNPs model were divided quarterly (Q1–Q4), the ranges were −1.73 to 2.71 in Q1, 2.71–4.76 in Q2, 5.01–7.43 in Q3, and 7.6–13.24 in Q4. The small SNPs model discriminated responders and non-responders; 27/28 (96.4%) in Q1 and 27/28 (96.4%) in Q4 were correctly predicted to be non-responders and responders, respectively (*p* < 0.0001, Fig. 1D).

**Table 3:** Important features in small SNPs model.

#	Parameter	Gene name	Score	Median weight
1	Diabetes mellitus		0.763	−0.026
2	Gleason score		0.659	−0.036
3	rs11231949	LOC101928443	0.561	−0.032
4	EOD grade		0.507	−0.025
5	rs2035081	PRIM1	0.432	−0.023
6	CK		0.224	0.032
7	Total P1NP		0.202	−0.029
8	Lymphocyte (%)		0.137	0.015

*CK* creatine kinase, *EOD* extent of disease, *P1NP* type I procollagen-N-propeptide, *SNP* single nucleotide polymorphism.

When the 46 SNPs (*p* < 1.0 × 10^−4^) were added to the clinical parameters, 4 clinical parameters and 19 SNPs were identified to be critical to predict castration resistance at 2 years (Table 4). Besides the known predictive factors, M-category and Gleason score, other factors including total bilirubin level and glucose level were also identified as critical parameters (Table 4). When the predictive model (large SNPs model) was created using the formula in Supplementary Table 2, the AUCs in the discovery and validation cohorts were prominently improved to 0.920 (95% CI, 0.854–0.986) and 0.978 (95% CI, 0.932–1.000), respectively (Fig. 1E). When scores calculated by the large SNPs model were divided quarterly (Q1–Q4), the ranges were 0.8439–5.716 in Q1, 5.834–7.5162 in Q2, 7.7932–9.5307 in Q3, 9.6555–14.3819 in Q4. The large SNPs model discriminated responders and non-responders; 19/21 (90.5%) in Q1 and 19/20 (95.0%) in Q4 were correctly predicted to be non-responder and responder, respectively (*p* < 0.0001, Fig. 1F).

**Table 4:** Important features in large SNPs model.

#	Parameter	Gene name	Score	Median weight
1	rs12979986	ZNF702P	0.988	−0.106
2	rs9625031	SRRD	0.968	−0.098
3	M-category		0.629	−0.038
4	rs1931229	VGLL2	0.512	−0.103
5	rs1660281	—	0.488	0.064
6	rs10064620	—	0.459	0.060
7	Gleason score		0.432	−0.066
8	rs10860210	RMST	0.395	−0.048
9	rs941207	BAZ2A	0.385	0.057
10	rs9868579	RPN1	0.317	−0.031
11	rs62174680	—	0.300	0.040
12	rs11232056	LOC101928443	0.259	0.040
13	rs8124833	C20orf78	0.251	0.037
14	rs74522810	—	0.234	−0.049
15	rs74369678	LRRN1	0.205	−0.036
16	NA (chr3:74213366)	—	0.190	0.104
17	rs2035081	PRIM1	0.188	−0.045
18	T-Bil		0.176	0.033
19	Glucose		0.149	−0.041
20	rs79404120	FAM19A5	0.146	0.058
21	rs11672661	COL5A3	0.129	−0.030
22	rs28625772	—	0.112	0.028
23	rs9298681	—	0.105	−0.036

*NA* not available, *SNP* single nucleotide polymorphism, *T-Bil* total bilirubin.

### Prognosis stratification by predictive models created by ML in advanced prostate cancer

**Fig. 2:**
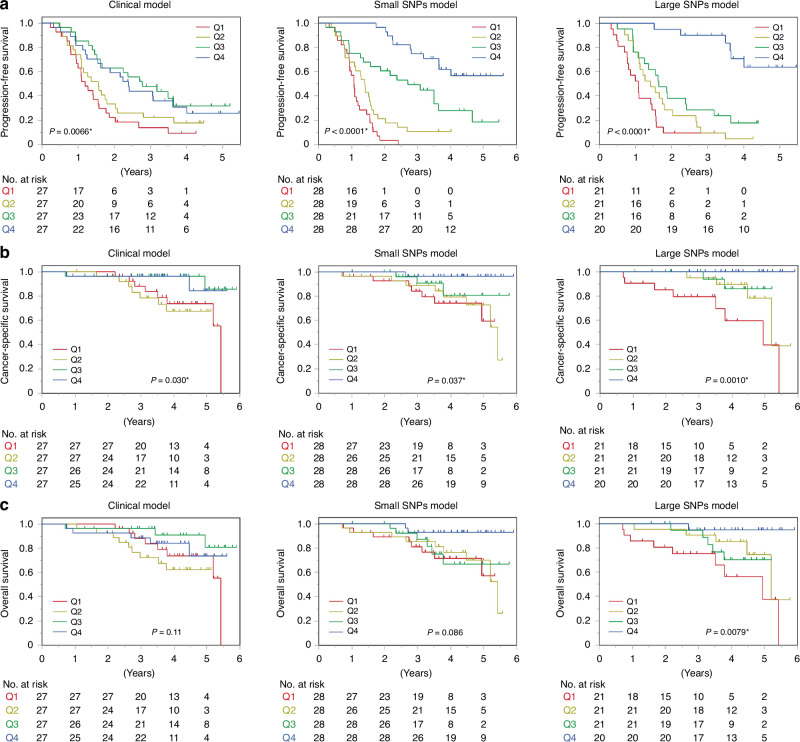
Prognosis stratification by predictive models of castration resistance at 2 years created by machine learning using clinical and genetic parameters. Progression-free survival (**A**), cancer-specific survival (**B**), and overall survival (**C**) among discovery and validation cohorts according to quarterly-divided groups (Q1–Q4) based on scores by clinical model (left), small SNPs model (middle), and large SNPs model (right).

We applied the three predictive models for prognosis stratification. PFS was significantly stratified by Q1–Q4 groups in all three models (Fig. 2A). The PFS was more prominently stratified in the small SNPs and large SNPs models than it was in the clinical model (Fig. 2A). The C-indices for PFS in the clinical, small SNPs, and large SNPs models were 0.617 (95% CI, 0.556–0.678), 0.727 (95% CI, 0.681–0.774), and 0.730 (95% CI 0.667–0.793), respectively (Supplementary Table 3). The CSS was also significantly stratified by the Q1–Q4 groups in all three models, but was stratified more prominently in the large SNPs model than it was in the clinical and small SNPs models (Fig. 2B). The C-indices for CSS in the clinical, small SNPs, and large SNPs models were 0.678 (95% CI, 0.546–0.809), 0.670 (95% CI, 0.551–0.790), and 0.781 (95% CI 0.671–0.890), respectively (Supplementary Table 3). The OS was significantly stratified by the Q1–Q4 groups only in the large SNPs model (Fig. 2C). The C-indices for OS in the clinical, small SNPs, and large SNPs models were 0.636 (95% CI, 0.520–0.753), 0.621 (95% CI, 0.512–0.731), and 0.703 (95% CI 0.583–0.822), respectively (Supplementary Table 3).

The preexisting well-known risk model, J-CAPRA risk group using TNM category, Gleason score, and PSA level stratified PFS, but not CSS and OS (Supplementary Fig. 1). The C-indices for PFS, CSS, and OS by J-CAPRA risk group were 0.588 (95% CI, 0.536–0.639), 0.602 (95% CI, 0.512–0.692), and 0.528 (95% CI 0.429–0.627), respectively (Supplementary Table 3).

### Allele frequency by ethnicity and estimated effect of important SNPs in the large SNPs model on the response to ADT

The allele frequency of SNPs is known to differ among ethnic populations, which may affect the impact of genotype on outcomes. We investigated the allele frequencies of important SNPs in the large SNPs model. Minor allele frequency data for 16 SNPs were available in the 1000 Genomes Project, and differed among different ethnic groups as shown in Table 5. We estimated the effect of critical SNPs in the large SNPs model on the response to ADT. The estimated effect of the 16 SNPs was 0.94 in East Asians and −1.20 in Europeans, where a high value indicates higher probability of responder (Table 5).

**Table 5:** Minor allele frequency by ethnics and estimated effect on the response to androgen deprivation therapy.

SNP identification	Coefficient	1000 Genome				
		SAS	AFR	EUR	AMR	EAS
rs12979986	−1	0.26	0.08	0.34	0.34	0.21
rs9625031	−1	0.14	0.02	0.29	0.19	0.23
rs1931229	−1	0.35	0.10	0.28	0.33	0.50
rs1660281	1	0.26	0.49	0.11	0.21	0.38
rs10064620	1	0.41	0.26	0.37	0.32	0.26
rs10860210	−1	0.45	0.50	0.38	0.46	0.32
rs941207	1	0.16	0.08	0.26	0.25	0.26
rs9868579	−1	NA	NA	NA	NA	NA
rs62174680	1	0.29	0.03	0.32	0.28	0.34
rs11232056	1	0.26	0.28	0.17	0.41	0.47
rs8124833	1	0.10	0.20	0.13	0.25	0.39
rs74522810	−1	0.20	0.18	0.21	0.18	0.18
rs74369678	−1	NA	NA	NA	NA	NA
NA (chr3:74213366)	1	NA	NA	NA	NA	NA
rs2035081	−1	0.22	0.33	0.38	0.33	0.26
rs79404120	1	0.17	0.01	0.14	0.11	0.16
rs11672661	−1	0.40	0.06	0.48	0.48	0.43
rs28625772	1	0.05	0.30	0.06	0.07	0.08
rs9298681	1	0.24	0.47	0.20	0.45	0.26
Estimated effect		−0.16	1.70	−1.20	0.08	0.94

*AFR* Africa, *AMR* native Americans, *EAS* East Asia, *EUR* Europe, *NA* not available, *SAS* South Asia, *SNP* single nucleotide polymorphism.

## Discussion

The results also showed higher predictive ability when SNPs were used in addition to clinical parameters. Several risk models using clinical parameters have been developed to predict the response and prognosis of ADT. However, their predictive power is modest, as indicated by AUCs of <0.7 [5–7]. We also found that the clinical model had limited predictive power even when created by ML, although the C-index was modestly higher than that of previous risk models. The predictive power of the models created by ML was improved by adding small and large numbers of SNPs to the clinical parameters. In particular, the large SNPs model achieved C-indices >0.70 for PFS, CSS, and OS. Considering various previous predictive models failed to achieve C-indices >0.70, achieving higher prediction power of castration resistance at 2 years and prognosis by measuring 19 SNPs in addition to four clinical parameters would be valuable. Currently, intensive treatments have emerged as novel standard treatments for advanced prostate cancer [2]. Therefore, the large SNPs model will be helpful in choosing the best treatment for individual patients. Intensive treatment may be preferable if patient is a non-responder while de-escalated treatment may be preferable if patient is predicted to be a responder.

In addition, genetic parameters in the large SNPs model supported the ethnic differences of response to ADT. Several studies reported that Asians have a higher survival rate after primary ADT compared with that of Caucasians and African Americans [8, 9, 27]. Consistently, the score from allele frequencies of 16 SNPs in the large SNPs model indicated a higher possibility of responders in East Asians compared with that in people with European ancestry.

We identified various clinical and genetic parameters that were associated with response to ADT. Among 19 SNPs in the large SNPs model, rs1931229 was associated with the expression of *TSPYL1*, which is known to be a CYP17A1 and CYP3A4 regulator, by expression quantitative trait loci (eQTL) analysis, whereas rs941207 and rs2035081 were associated with *HSD17B6* expression (data not shown). Because *TSPYL1* and *HSD17B6* are both involved in androgen synthesis, they are associated with the response to ADT through their role in this pathway [28, 29]. Preexistence of hypertension was associated with favorable response to ADT, whereas preexistence of diabetes mellitus and high glucose level were associated with unfavorable response to ADT. This is consistent with our previous finding that comorbidity with hypertension and diabetes mellitus were associated with longer and shorter survival in primary ADT, respectively [30, 31]. Higher total cholesterol level was also associated with better response to ADT, whereas preexistence of dyslipidemia was associated with poor response to ADT, implying a close relationship between ADT and lipid metabolism.

This study had several limitations. The sample size was relatively small. Although the SNPs models had excellent predictive performance in the Japanese population, future work is needed to explore the generalizability of the predictive performance of these SNPs models in other populations. Primary ADT alone is no longer a standard therapy, and utilized in a combination with other treatments such as androgen receptor signaling inhibitors. Conversely, a strong point of this study is that the clinical data were obtained from patients enrolled in a prospective trial, in which the treatment and testing schedules were subject to strict protocols.

## Conclusion

Our results demonstrate that the SNPs models using clinical and genetic parameters created by a PWL algorism produced excellent prediction of castration resistance and prognosis in primary ADT for advanced prostate cancer. These models are expected to be helpful in treatment choice for advanced prostate cancer.

## Supplementary information

Supplementary Figure 1

Supplementary Table 1

Supplementary Table 2

Supplementary Table 3

## Data Availability

The data sets generated and/or analyzed during the current study are available from the corresponding author upon reasonable request.
