# Supplementary material for: Predictive model of castration resistance in advanced prostate cancer by machine learning using genetic and clinical data: KYUCOG-1401-A study
Source: BJC Rep. 2024 Sep 9;2:69. doi: 10.1038/s44276-024-00093-3 (PMC11523954; doi:10.1038/s44276-024-00093-3)
Supplement: Supplementary file 1 — Supplementary Figure 1 [file 44276_2024_93_MOESM1_ESM.pptx]

## Slide 1
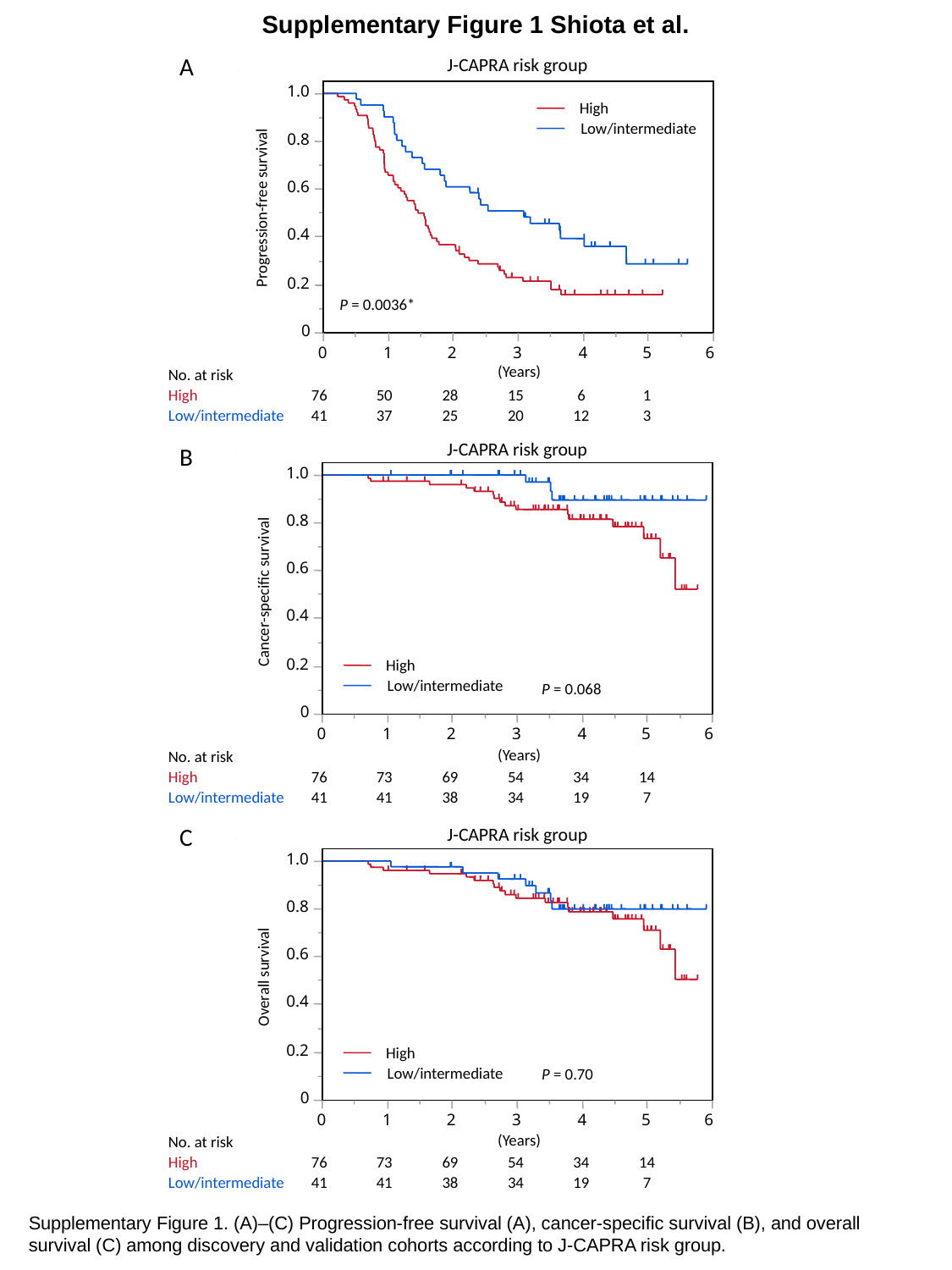

Supplementary Figure 1 Shiota et al.
A
J-CAPRA risk group
1.0
0.8
0.6
0.4
0.2
0
0
1
2
3
4
5
6
High
Low/intermediate
Progression-free survival
P = 0.0036*
(Years)
| No. at risk | | | | | | |
| --- | --- | --- | --- | --- | --- | --- |
| High | 76 | 50 | 28 | 15 | 6 | 1 |
| Low/intermediate | 41 | 37 | 25 | 20 | 12 | 3 |
B
J-CAPRA risk group
1.0
0.8
0.6
0.4
0.2
0
0
1
2
3
4
5
6
Cancer-specific survival
High
Low/intermediate
P = 0.068
(Years)
| No. at risk | | | | | | |
| --- | --- | --- | --- | --- | --- | --- |
| High | 76 | 73 | 69 | 54 | 34 | 14 |
| Low/intermediate | 41 | 41 | 38 | 34 | 19 | 7 |
C
J-CAPRA risk group
1.0
0.8
0.6
0.4
0.2
0
0
1
2
3
4
5
6
Overall survival
High
Low/intermediate
P = 0.70
(Years)
| No. at risk | | | | | | |
| --- | --- | --- | --- | --- | --- | --- |
| High | 76 | 73 | 69 | 54 | 34 | 14 |
| Low/intermediate | 41 | 41 | 38 | 34 | 19 | 7 |
Supplementary Figure 1. (A)–(C) Progression-free survival (A), cancer-specific survival (B), and overall survival (C) among discovery and validation cohorts according to J-CAPRA risk group.
